# Stress Tolerance and Symbiotic and Phylogenic Features of Root Nodule Bacteria Associated with *Medicago* Species in Different Bioclimatic Regions of Tunisia

**DOI:** 10.1264/jsme2.ME10138e

**Published:** 2020-09-05

**Authors:** Salem Djedidi, Tadashi Yokoyama, Naoko Ohkama-Ohtsu, Chandra Prasad Risal, Chedly Abdelly, Hitoshi Sekimoto

**Affiliations:** 1 United Graduate School of Agricultural Science, Tokyo University of Agriculture and Technology, 3–5–8 Saiwai-cho, Fuchu, Tokyo 183–8509, Japan; 2 Institute of Agriculture, Tokyo University of Agriculture and Technology, 3–5–8 Saiwai-cho, Fuchu, Tokyo 183–8509, Japan; 3 Women’s Future Development Organization, Tokyo University of Agriculture and Technology, 3–5–8 Saiwai-cho, Fuchu, Tokyo 183–8509, Japan; 4 Laboratory of Plant Adaptation to Abiotic Stresses (LAPSA), Biotechnology Centre of Borj Cedria, P.O. Box 901, 2050 Hammam-Lif, Tunisia; 5 Faculty of Agriculture, Utsunomiya University, 350 Mine, Utsunomiya, Tochigi 321–8505, Japan

Vol. 26, No. 1, 36–45, 2011

 

p. 40 right column

**Incorrect**

St.3-13, Pl.2-1 and Pl.3-6 categorized as *A. tumefaciens* displayed acetylene reduction activities which were not significantly different in comparison with the remaining isolates classified into *E. meliloti* or *E. medicae*, except for St.3-1 as shown in Fig. 6-B.

**Correct**

St.3-13, Pl.2-1 and Pl.3-6 categorized as *A. tumefaciens* displayed acetylene reduction activities as shown in Fig. 6-B.

 

p. 43

**Incorrect**

Fig. 6 A and B with the small letters, a, ab, b mistakenly placed on the bar plots and indicating statistical analysis

**Correct**

Fig. 6 A and B without the small letters, as below

**Fig. 6.**
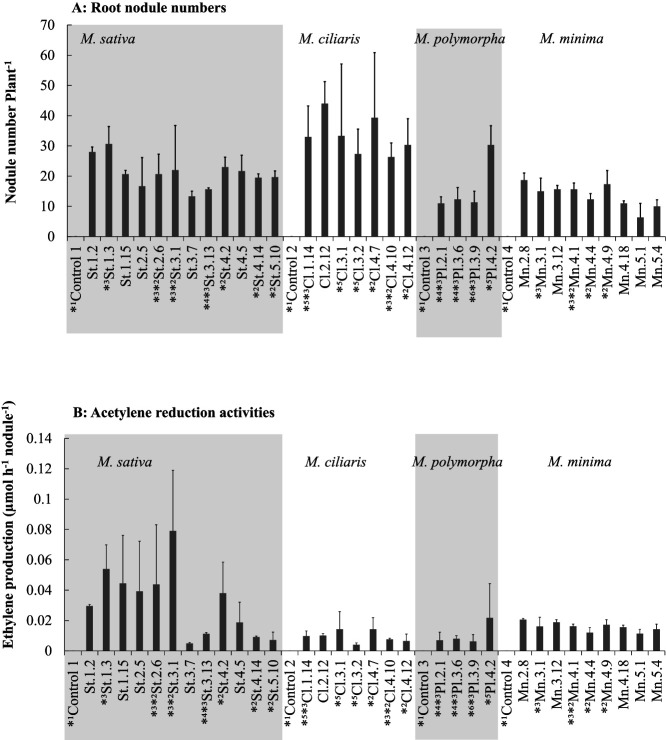
Root nodule numbers and Acetylene reduction assay of *Medicago* plants inoculated with the 32 isolates. *1: uninoculated control plants, *2: isolates with high temperature tolerance, *3: isolates with high salinity tolerance, *4: isolates categorized as *A. tumefaciens*, *5: isolates assigned to *E. medicae*, *6: isolate categorized as *E. meliloti*; however, the sequence of nodA was identical to that of *E. medicae*.

p. 44 left column

**Incorrect**

This result shows that the root nodule forming ability of the symbiotic *Agrobacterium* is significantly lower than that of the *E. medicae* isolate.

**Correct**

This result shows that the root nodule forming ability of the symbiotic *Agrobacterium* is lower than that of the *E. medicae* isolate.

 

p. 44 left column

**Incorrect**

Similarly, regarding ethylene production by acetylene reduction assays, those of the two *Agrobacterium* isolates were clearly lower than that of *E. medicae* isolates shown in Fig. 6-B.

**Correct**

Similarly, regarding ethylene production by acetylene reduction assays, those of the two *Agrobacterium* isolates were lower than that of *E. medicae* isolates shown in Fig. 6-B.

